# Recent Advances in Lithium Ion/Lithium Metal Hybrid Anodes: from Design Principles to Practical Applications

**DOI:** 10.1002/advs.202514517

**Published:** 2025-11-08

**Authors:** Xianze Yin, Huan Ye, Zi‐Jian Zheng

**Affiliations:** ^1^ College of Materials Science and Engineering State Key Laboratory of New Textile Materials & Advanced Processing Technology Wuhan Textile University Wuhan 430073 China; ^2^ College of Chemistry Huazhong Agricultural University Wuhan 430070 China; ^3^ Ministry of Education Key Laboratory for the Green Preparation and Application of Functional Materials Hubei Key Laboratory of Polymer Materials Hubei University Wuhan 430062 China

**Keywords:** high energy density, hybrid anode, lithium metal, lithium metal battery, stability

## Abstract

Lithium metal is widely regarded as the ultimate anode material for next‐generation high‐energy‐density batteries due to its exceptional theoretical specific capacity (3860 mA h g^−1^) and low redox potential (−3.04 V vs standard hydrogen electrode). However, its practical application is hindered by low Coulombic efficiency, rapid capacity degradation, and severe safety risks caused by uncontrolled dendrite growth, substantial volume variations, and unstable solid electrolyte interphase formation. To address these limitations, an emerging paradigm involves the strategic integration of lithium metal with porous graphite or graphitized carbon hosts, forming lithium‐ion/lithium‐metal hybrid anodes. Such hybrid architectures utilize the synergistic advantages of both materials: graphite provides a mechanically robust, conductive framework with a well‐defined structure to regulate Li plating/stripping processes, while Li metal delivers unparalleled capacity. This review systematically summarizes recent breakthroughs in mechanistic understanding, configuration designs, and electrolyte engineering that optimize the performance of hybrid anodes for high‐energy lithium metal batteries. A critical analysis of the interfacial stabilization and the influence of electrolyte composition in improving cycling stability is conducted. Finally, a concise conclusion and prospective outlook regarding current challenges and future research opportunities in the material design and the development of compatible electrolyte systems of hybrid anode systems are proposed.

## Graphical abstract

Lithium‐graphite hybrid anodes offer a promising solution to the challenges of lithium metal anodes, such as dendrite growth and instability. By combining graphite's stable framework with lithium's high capacity, these anodes enable controlled plating and enhance safety. This review examines their design principles, mechanistic insights, and compatible electrolytes, connecting fundamental research to practical applications for next‐generation high‐energy batteries.

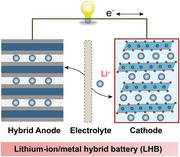

## 1 Introduction

The demand for high energy density and enhanced safety has been a central focus in battery development, where progress predominantly depends on innovations in electrode materials.^[^ ^1^, ^2^, ^3^, ^4^, ^5^ ^]^ Graphite has emerged as the most promising anode material for rechargeable lithium‐ion batteries (LIBs) due to its competitive advantages, including high theoretical capacity, suitable working potential, dimensional stability, and cost‐effectiveness.^[^ ^6^, ^7^, ^8^, ^9^ ^]^ However, due to the inherent limitations of intercalation chemistry, graphite can only deliver a theoretical specific capacity of 372 mAh g^−1^, which is insufficient for advancing energy density in practical applications. In efforts to enhance the energy density of LIBs, significant research has been devoted to developing novel high‐capacity anode materials.^[^ ^10^, ^11^ ^]^ Among these, Li metal stands out as the “holy grail” anode material of rechargeable Li batteries due to its impressive specific capacity of 3860 mAh g^−1^, and a low reduction potential of −3.04 V versus the standard hydrogen electrode. Li metal operates through a reversible plating/stripping mechanism that facilitates the transformation between chemical energy and electronic energy.^[^ ^12^, ^13^, ^14^ ^]^ The concept of rechargeable Li metal batteries (LMBs) dates back to the 1970s with the pioneering work on Li‐TiS_2_ cells by Exxon.^[^ ^15^ ^]^ However, this promising battery technology was later withdrawn from the market due to underwhelming cycling performance, primarily attributed to dendritic Li growth and the formation of an unstable solid electrolyte interphase (SEI) during prolonged cycling.^[^ ^16^, ^17^, ^18^ ^]^ The resistive nature of the SEI leads to uneven current distribution across the Li anode surface, exacerbating the growth of Li dendrites. The continuous plating and stripping of these dendrites significantly increase both Li consumption and the depletion of the liquid electrolyte, thereby lowering Coulombic efficiency and Li utilization. Additionally, dendritic Li presents two major concerns: it can either penetrate the separator and create an internal short circuit by reaching the cathode, or it may break off, losing electronic contact with the conductive substrate, resulting in so‐called “dead Li”.^[^ ^19^, ^20^ ^]^ This chemically active but electrochemically inactive Li leads to reduced Li utilization and a shortened battery lifespan. Moreover, Li metal undergoes infinite volume changes due to its intrinsic “hostless” nature.^[^ ^21^, ^22^ ^]^ Such characteristics prevent the formation of a robust and stable SEI film, further exacerbating the challenges associated with Li metal anodes.

Embedding Li metal in porous substrates is considered an effective strategy to address various issues faced by rechargeable Li batteries in practical applications.^[^ ^23^, ^24^, ^25^, ^26^ ^]^ Currently, a variety of 3D porous materials, including 3D metallic current collectors^[^ ^27^, ^28^, ^29^ ^]^ and carbon‐based current collectors,^[^ ^30^, ^31^, ^32^ ^]^ have been developed as hosts for Li metal. These porous hosts exhibit high specific area and sufficient active sites, enabling localized electric field redistribution and ensuring low local current densities, which promote homogeneous nucleation and growth of Li. Additionally, the porous structure can serve as a reservoir for Li, mitigating the substantial volume changes that occur during charge and discharge cycles. Despite these advantages, the complexity of fabrication processes and the associated high costs currently limit the application of these composite Li metal anodes.^[^ ^33^, ^34^ ^]^ In addition, these 3D porous architectures may significantly reduce the overall volumetric energy density due to their substantial space occupation within the cell configuration. Graphite and graphitized carbons, well‐established materials, have shown potential as effective hosts for Li metal.^[^ ^32^, ^35^ ^]^ It not only effectively controls the deposition of Li metal but also enables the formation of a hybrid anode composed of both Li metal and graphite/graphitized carbons. By incorporating small amounts of Li within the graphite, significant suppression of overall electrode volume expansion is achieved. Furthermore, this hybrid anode demonstrates considerable improvements in volumetric energy density by 25% and gravimetric energy density by 20% compared to conventional LIBs, offering promising advancements for the future development of lithium battery technology.

**Figure 1:**
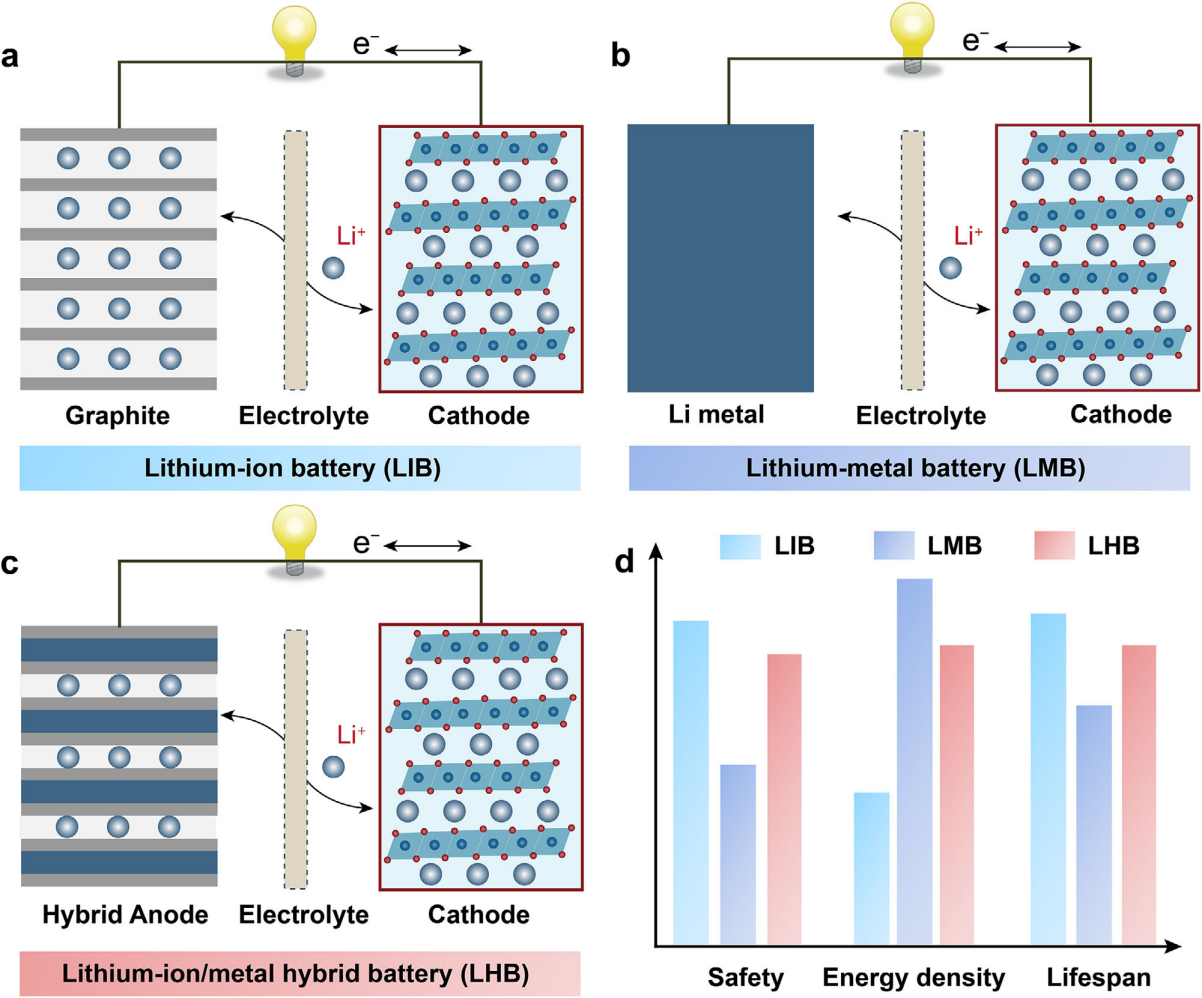
Schematic illustrations and comparison of lithium‐based battery systems. a) Working mechanism of a lithium‐ion battery (LIB). b) Working mechanism of a lithium‐metal battery (LMB). c) Working mechanism of a lithium‐ion/metal hybrid battery (LHB). d) Comparative analysis of the advantages and disadvantages of LIB, LMB, and LHB.

However, there remain knowledge gaps in comprehensive studies aimed at investigating the mechanisms underlying Li metal deposition on graphite/graphitized carbons and the electrochemical characteristics influencing this process across various graphite materials. Furthermore, the electrochemical behavior of hybrid anodes varies significantly depending on electrolyte composition, yet no consensus exists on whether LIB‐optimized or LMB‐optimized electrolytes are more suitable. In this review, we aim to bridge these gaps by providing a comprehensive analysis of recent progress in lithium‐ion/lithium‐metal hybrid anodes. We will systematically analyze their working mechanisms alongside the structure–performance relationships (**Figure** **1**). Moreover, we will present perspectives and suggestions aimed at optimizing graphite/graphitized carbons and electrolyte formulations to enhance the electrochemical performance and practical applicability of rechargeable Li batteries.

## 2 Working Mechanism of Hybrid Anodes

**Figure 2:**
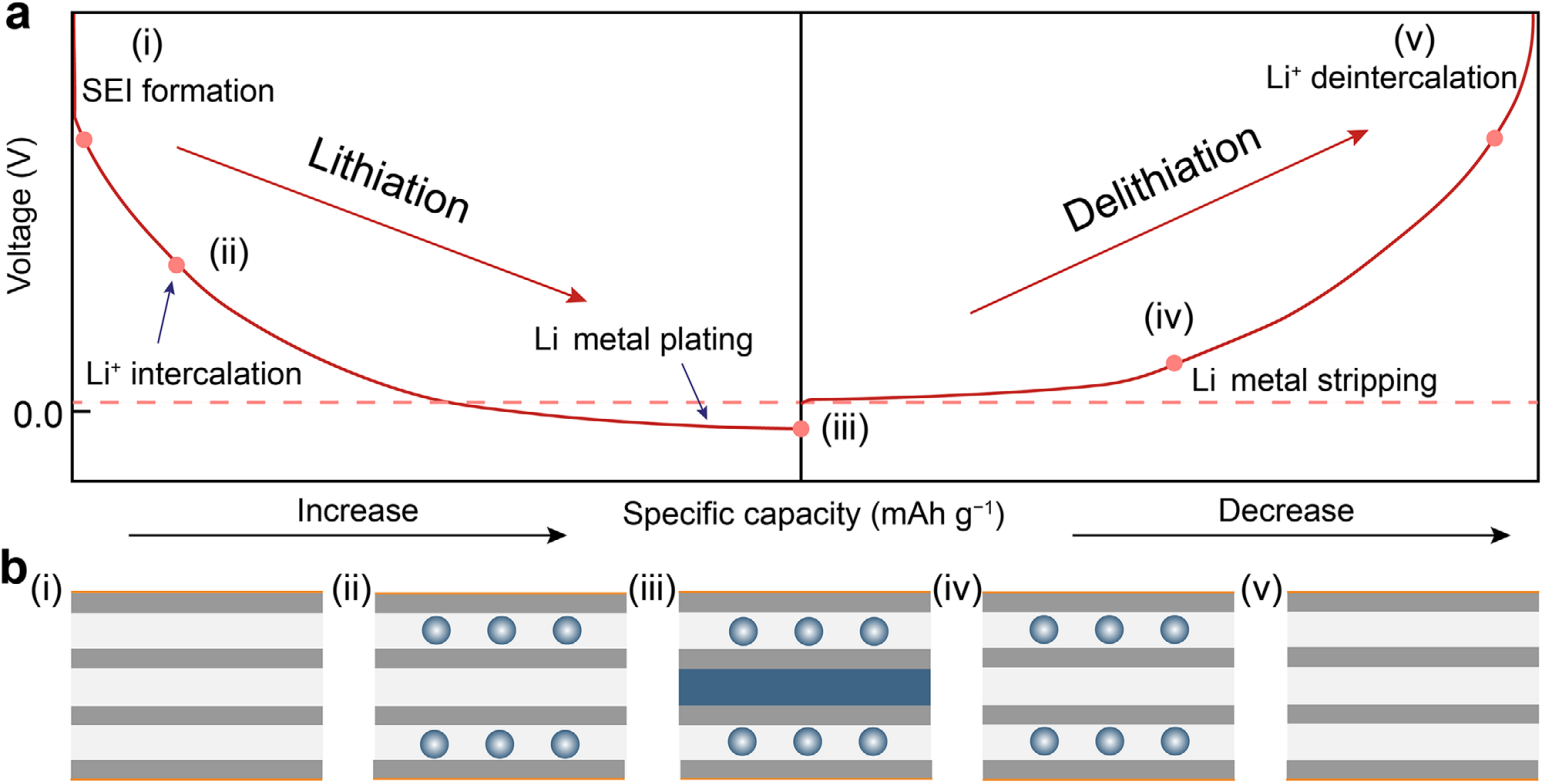
Reaction mechanisms and electrochemical performance of a lithium‐ion/metal hybrid anode. a) Voltage profiles during charge/discharge cycles. b) Schematic of Li⁺ migration and phase evolution during cycling.

The lithium‐ion/lithium‐metal hybrid anode achieves a balance between high capacity and long cycling life in rechargeable Li batteries through the synergistic effects of lithium‐ion intercalation/deintercalation reactions and lithium metal plating/stripping processes.^[^ ^36^ ^]^ The hybrid system undergoes five distinct yet interconnected electrochemical stages during cycling (**Figure** **2**): i) formation of the solid electrolyte interphase (SEI) on the graphite surface, ii) Li^+^ intercalation into the graphene layers, iii) plating of Li metal on the graphite substrate, iv) stripping of Li metal from the graphite, and (v) Li^+^ deintercalation from graphene layers. Each stage plays a critical role in stabilizing the electrode–electrolyte interface and mitigating the degradation of electrochemical performance. During the initial charging stage, lithium ions deintercalate from the cathodes and migrate toward the graphitic carbon anode, forming the intercalation compound Li_x_C_6_ (x≤1). This process is accompanied by charge transfer from the Li 2s orbitals to unoccupied π* antibonding orbitals of graphite, leading to partial electron delocalization across the carbon layers.^[^ ^37^ ^]^ The resulting negatively charged graphene sheets exhibit enhanced affinity for Li and may contribute to a uniform Li^+^ flux. Furthermore, the elevated Fermi level caused by the presence of filled 2s electrons from lithium atoms improves the electronic conductivity within the intercalation compound, which is crucial for the reversible plating/stripping of Li metal. When the anode potential falls to the lithium deposition threshold below 0 V (vs Li⁺/Li), excess lithium ions undergo electrochemical reduction on the pre‐formed Li_x_C_6_ surface. The electron‐rich carbon matrix effectively redistributes interfacial current density, leading to the generation of compact Li deposits characterized by a uniform, non‐dendritic morphology.

During battery discharging, the deposited Li metal is stripped from the interfaces of the graphite. The highly conductive network established by graphite ensures that metallic Li can reversibly dissolve while maintaining the dynamic stability of the hybrid anode volume. During a high discharge state, lithium ions stored in the graphite are gradually released, which not only compensates for the active lithium lost during cycling but also contributes to the stabilization of the SEI layer through controlled volume changes. This multi‐stage coupling mechanism provides a complementary advantage between lithium‐ion storage and Li metal deposition, addressing the failure issues associated with traditional Li metal anodes. By utilizing these synergistic processes, the lithium‐ion/lithium‐metal hybrid anode presents a promising avenue for enhancing the performance and reliability of next‐generation Li batteries.

## 3 Hybrid Anode Design

### 3.1 Graphite‐Based Hosts

According to the structural difference, carbons can roughly be divided into graphitic and non‐graphitic.^[^ ^38^ ^]^ Graphitic carbons refer to carbonaceous materials that have a relatively perfect layered structure. Non‐graphitic carbons with a disordered structure are composed of an amorphous phase and a crystalline phase. Graphite, consisting of sp^2^ hybridized graphene layers with a stacking order either as ABAB… (α‐hexagonal) or ABCABC… (β‐rhombohedral) configuration, belongs to graphitic carbon. Two neighboring graphene layers with an interlayer spacing of 0.335 nm enable reversible intercalation–extrusion of Li^+^ into the graphite host, providing its good cyclability and safety for LIB anodes.^[^ ^39^ ^]^ Owing to the delocalized π‐bonds, graphite usually shows high electronic (in‐plane) conductivity, which is in favor of the rapid electron transfer.^[^ ^40^ ^]^ However, due to the occupation of Li between two adjacent graphene planes, the maximum composition of lithium intercalated graphite (LiC_6_) can be achieved to yield a usable specific capacity of 372 mAh g^−1^. Furthermore, the intercalation mechanism imposes kinetic barriers at high current densities, while the low equilibrium potential (0.1 V vs Li^+^/Li) increases the risk of parasitic reactions and unintended Li metal plating outside of the graphite structure.^[^ ^41^, ^42^ ^]^ In contrast, these inherent characteristics of graphite enable it as an exceptional host for Li metal anode since its ordered graphene layers provide geometrically confined spaces that regulate Li nucleation and suppress dendrite growth, while the high conductivity of graphite ensures efficient charge distribution and rapid charge transfer throughout the anode. The structural integrity of graphite is also noteworthy, as it experiences minimal volume changes of less than 10% during lithium‐ion intercalation/deintercalation, thereby accommodating the volume fluctuations that occur during battery cycling. Additionally, its chemical compatibility with conventional LIB electrolytes offers significant practical advantages compared to bare Li metal, reducing risks associated with reactivity and expanding its compatibility with various high‐voltage cathodes.

**Figure 3:**
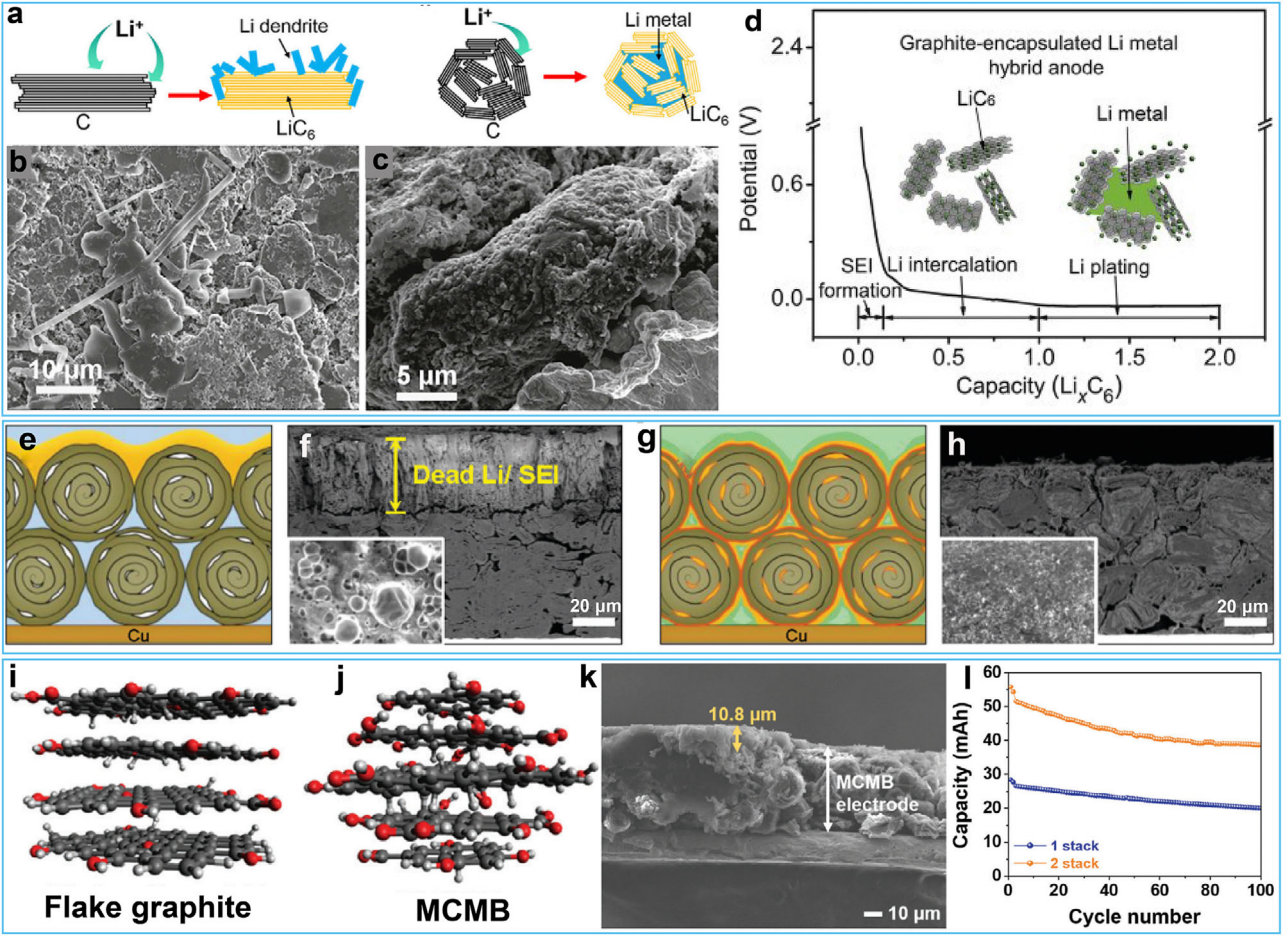
a) Li deposition mechanisms on 2D graphite flakes and massive artificial graphite (MAG) particles. b, c) SEM images of Li‐coated flakes and MAG particles at double the capacity of traditional graphite intercalation anodes. d) Deposition voltage profile and schematic morphology of Li‐MAG composites. Reproduced with permission.^[^ ^43^ ^]^ Copyright 2016, Elsevier. e, f) Schematic morphology and SEM image of Li‐graphite composites with liquid electrolyte after 20 cycles. g, h) Schematic morphology and SEM image of Li–Si embedded graphite composites with gel electrolyte after 50 cycles. Reproduced with permission.^[^ ^47^ ^]^ Copyright 2020, The Royal Society of Chemistry. i, j) Ball‐and‐stick models of flake graphite and mesocarbon microbeads (MCMB) particles. k) Cross‐sectional SEM images of MCMB electrodes after the 10th charge (intercalation: plating = 1:1.5). l) Cycling performance comparison of pouch cells containing either single‐layer or double‐layer stacked electrodes. Reproduced with permission.^[^ ^49^ ^]^ Copyright 2024, Wiley‐VCH.

Cui and co‐workers investigated the use of both natural graphite, characterized by large 2D flakes, alongside synthetic graphite, which is composed of a 3D packing of small 2D graphite flakes into a secondary particle, as host materials for the confined deposition of Li metal (**Figure** **3**a).^[^ ^43^ ^]^ Their study revealed distinct deposition behaviors depending on the graphite morphology. In natural graphite with exposed 2D flakes, Li nucleation preferentially occurred at the edge planes due to their higher electrochemical activity compared to basal planes,^[^ ^44^, ^45^, ^46^ ^]^ leading to dendritic Li growth on the large 2D graphite sheets (Figure 3b). In contrast, massive artificial graphite (MAG) particles, characterized by basal‐plane‐dominated surfaces and an interior structure rich in edge planes with void spaces, enabled Li deposition primarily within the internal voids (Figure 3c). This confined Li deposition effectively suppressed Li dendrite formation both on the surface and inside the electrodes, while simultaneously reducing undesirable side reactions with the electrolyte. Galvanostatic charge–discharge profiles coupled with ex situ X‐ray diffraction (XRD) analysis elucidated stepwise electrochemical processes (Figure 3d): i) an initially short sloping plateau at ∼0.7 V corresponding to the formation of SEI, ii) several potential plateaus between 0.7 and 0.01 V representing Li intercalation into the graphene layers, and iii) a long potential plateau at −0.01 V indicating Li metal plating within the graphite electrode. By precisely controlling the plating time, they achieved tunable Li deposition capacities ranging from 744 mAh g^−1^ to 1166 mAh g^−1^, demonstrating the potential of the hybrid anode for capacity optimization. However, due to the limited internal void space within the MAG host, high‐areal‐capacity Li deposition leads to significant metal accumulation on the electrode surface. This exposed metallic Li readily reacts with carbonate‐based electrolytes, resulting in a reversible Coulombic efficiency of only 96.6% and a severely limited cycle life of merely 30 cycles.

Recently, Volder and colleagues synthesized low‐surface‐area graphite spherical particles with high electrode density through the impact milling of commercial natural graphite.^[^ ^47^ ^]^ The resulting porous graphite exhibited a well‐defined structure with a porosity of 36% and an average pore size of 300–500 nm, providing sufficient void space to accommodate a remarkable Li storage capacity of 963 mAh g^−1^. To mitigate undesirable side reactions and suppress Li dendrite formation induced by defective sites on the graphite surface, the researchers employed chemical vapor deposition to coat the graphite with a conformal amorphous Si layer with an ≈15 nm thick. The results indicated that after 20 cycles, the pristine graphite was heavily passivated by 30 µm of by‐products, including dead Li and SEI layers, whereas the Si‐coated graphite showed no sign of excessive “dead Li” or SEI accumulation (Figure 3e–h). Despite these merits, practical challenges persist when integrating the graphite/Li hybrid anode into full cells. Even with exceptional capacity retention over 200 cycles at designated capacities of either 600 or 800 mAh g^−1^ caused by the well‐confined Li in the voids of graphite, the suppression of side reactions, and the reduction of inactive Li, full cells paired with commercial LiCoO_2_ cathodes showed rapid capacity fading by presenting 74.5% capacity retention after 50 cycles. This performance gap likely arises from multifaceted factors, including a relatively low cathode loading, high N/P ratios, and inadequate electrolyte compatibility. Kang et al. developed a spherical graphite host material through chemical lithiation and controlled expansion of natural spherical graphite (NSG).^[^ ^48^ ^]^ This engineered expansion created a hierarchically porous structure that simultaneously enhanced lithium‐ion transport kinetics and provided confined spaces for homogeneous lithium deposition. The resulting anode exhibited exceptional electrochemical performance, delivering a reversible hybrid capacity of 558 mAh g^−1^ through optimized intercalation/deintercalation and plating/stripping processes. When paired with a high‐loading NCM811 cathode (13 mg cm^−2^) in conventional carbonate electrolytes, the full‐cell configuration demonstrated remarkable cycling stability, maintaining a Coulombic efficiency of 99.81% over 200 cycles even at a low N/P ratio of 1.15.

To address these limitations, Choi et al. employed mesocarbon microbeads (MCMB) as a host material for the LIB/LMB hybrid system.^[^ ^49^ ^]^ The MCMB featured a spherical morphology, abundant lithiophilic functional groups (such as ─OH and C─O), and substantial interparticle space characterized by a porosity of 41% (Figure 3i,j). These features shortened Li‐ion diffusion pathways, enhanced the affinity of lithium ions for the MCMB, and lowered the effective current density, thereby enabling uniform Li deposition within the voids and ensuring reversible Li cycling at the hybrid anode (Figure 3k). When paired with LiNi_0.8_Co_0.1_Mn_0.1_O_2_ (NCM811) cathodes with an areal capacity of 4 mAh cm^−2^ in a localized high‐concentration electrolyte (2 m LiFSI in BTFE/DME), hybrid anodes with intercalation‐to‐plating capacity ratios of 2:1, 1:1, and 1:2 showed a capacity retention of 83.5, 73.5, and 53.7% after 200 cycles, respectively. To alleviate the battery performance degradation caused by the pore blockage by upper Li deposition, the researchers further introduced a non‐conductive poly(vinylidene fluoride) (PVDF) polymer with a high dielectric constant as the top layer of the hybrid electrode. LiNi_0.8_Co_0.15_Al_0.05_O_2_ (NCA)‐paired pouch cells with a high areal capacity of 4.78 mAh cm^−2^ and a PVDF interfacial layer delivered a high energy density of 1062.3 Wh L^−1^ with a single stacked electrode layer and 1101.0 Wh L^−1^ with two stacked layers, showcasing the application potential of this hybrid configuration. The pouch cells, featuring one stack and two stacks, retained 75.5% and 74.9% of their original capacity after 100 cycles, respectively (Figure 3l). However, the use of highly concentrated electrolytes not only limits the scenarios in which the hybrid anode can be used but also increases the production costs of the battery.

Recently, Li et al. developed an ED@G@Ag Janus electrode featuring an ethylene‐propylene‐diene monomer (EPDM) insulating copolymer on the upper graphite surface, while retaining a lithiophilic Ag nanoparticle layer on the opposite side.^[^ ^50^ ^]^ The electronically insulating and lithiophobic EPDM, characterized by its nonpolar aliphatic chain, effectively suppressed Li^+^ reduction on the graphite anode's top surface and elevated the thermodynamic barrier for metallic lithium nucleation. Meanwhile, the lithiophilic Ag nanoparticle layer deposited on the Cu current collector functioned as preferential nucleation sites, guiding Li deposition predominantly beneath the graphite layer. This asymmetric design enabled LiFePO_4_‐based full‐cells to achieve a high‐capacity retention of 75% over 500 cycles at 1 C with a limited N/P ratio of 1.9. Based on this concept, the same group employed a facile spray‐coating technique to deposit an insulating ethylene vinyl alcohol copolymer (EVOH) film onto the surface of a graphite anode (Gra‐OH).^[^ ^51^ ^]^ The EVOH layer effectively suppressed electron migration across the graphite surface, thereby confining excessive lithium deposition within the porous graphite matrix. This hybrid Gra‐OH anode exhibited a high lithium storage capacity of 600 mAh g^−1^ while maintaining superior Coulombic efficiencies in both coin‐cell and pouch‐cell configurations, even when using conventional carbonate‐based electrolytes.

Further optimization was achieved using a porous graphite layer (PGL) synthesized via an industry‐scalable spontaneous template removal process from a mixture of graphite, NH_4_HCO_3_, carbon black, and PVDF.^[^ ^52^ ^]^ When it was used as a Li host, the PGL electrode delivered an ultrahigh Coulombic efficiency of 99.5% for 180 cycles even with a 30% excess of plated Li at 1 mA cm^−2^. This performance can be attributed to the high reversibility of Li from a continuous dissolution–deintercalation mechanism. In a hybrid LIB/LMB full cell utilizing prelithiated cathode and Li‐free PGL anode with a P/N ratio of 1.3 (the capacity ratio of NCM811 to graphite), stable cycling with a capacity retention of 67.2% under a practical cathode capacity of 3 mAh cm^−2^ was maintained for 300 cycles. In the future, further exploration of novel electrolytes to enhance their compatibility with high‐capacity electrode materials and to mitigate side reactions will be encouraged. Additionally, the design of innovative interfacial modification layers aimed at suppressing Li dendrite growth and minimizing the irreversible accumulation of SEI layers will be greatly welcomed.

**Figure 4:**
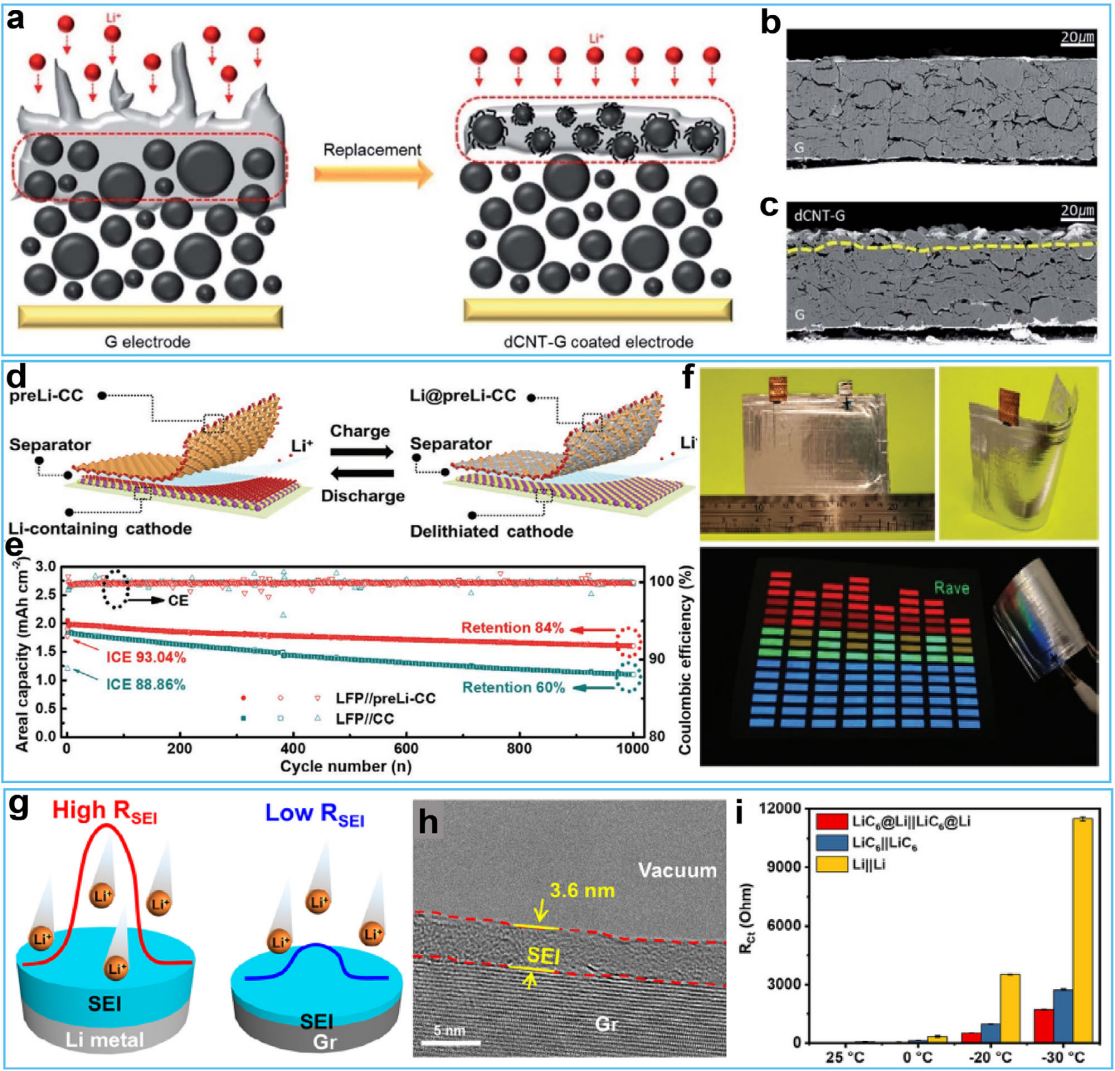
a) Schematic illustration of G electrode and dCNT‐G coated electrode. b, c) Cross‐sectional SEM images of G electrode and dCNT‐G coated electrode. Reproduced with permission.^[^ ^53^ ^]^ Copyright 2022, The Royal Society of Chemistry. d) Operation mechanism of flexible Li‐ion/Li‐metal hybrid batteries. e) Cycling performance comparison of LiFePO_4_//preLi‐CC and LFP//CC batteries. f) Photographic demonstration of flexible pouch cell performance: flat configuration, bent state, and powering an LED T‐shirt while curved. Reproduced with permission.^[^ ^58^ ^]^ Copyright 2022, Wiley‐VCH. g) Comparative schematics illustrating the Li^+^ migration mechanisms through the native SEI on lithium metal, as opposed to the structured SEI on a graphite (Gr) anode. h) TEM image of the SEI formed on Gr electrode. i) Electrochemical impedance spectra of symmetrical cells: Li||Li, LiC_6_||LiC_6_, and LiC_6_@Li||LiC_6_@Li measured at 25—30 °C. Reproduced with permission.^[^ ^59^ ^]^ Copyright 2025, Wiley‐VCH.

In addition to the conventional graphitic carbon materials mentioned above, recent advancements have introduced an expanded graphite, a defective‐carbon nanotube (dCNT)‐grown graphite (dCNT‐G) composite material, and a prelithiated carbon cloth as two host materials for LIB/LMB hybrid anodes. For instance, Kim and co‐workers have developed a simple chemical vapor deposition (CVD) using hydrogen (H_2_) and ethylene (C_2_H_4_) as precursor gases to fabricate a defective‐carbon nanotube (dCNT)‐grown graphite (dCNT‐G) composite to address the critical challenges of limited capacity for conventional graphite and dendritic growth for Li metal (**Figure** **4**a–c).^[^ ^53^ ^]^ Because of its defective structures with highly reactive sites, these dCNT regulated a dense and dendrite‐free Li deposition on the surface of the dCNT‐G electrode. When evaluated in a full‐cell configuration with a low N/P ratio of 0.8, the Li–dCNT‐G hybrid anode demonstrated exceptional cycling stability, maintaining 83.5% of its capacity after 300 cycles in conventional carbonate electrolytes. This performance represented a 13.5% improvement compared to a conventional Li‐graphite (Li‐G) hybrid anode under identical conditions. However, despite its promising electrochemical performance, the practical implementation of dCNT‐G composites faces challenges due to the limitations of the current preparation method. The CVD process employed for synthesizing dCNT‐G, while effective in producing defective nanostructures with controlled Li deposition behavior, presents scalability issues for industrial‐scale production.

For flexible battery applications, carbon‐based current collectors, such as nanotubes, graphene, and carbon cloth (CC), are increasingly utilized in the design and fabrication of high‐performance electrodes.^[^ ^54^, ^55^, ^56^, ^57^ ^]^ In particular, Zheng et al. reported a flexible hybrid LIB/LMB (f‐LIMB) using prelithiated CC (preLi‐CC) as a host, which uniquely incorporated both intercalation/deintercalation and plating/stripping mechanisms.^[^ ^58^ ^]^ The prelithiation process not only forms lithiophilic Li_x_C_6_ compounds to facilitate uniform Li deposition but also acts as a Li reservoir to compensate for irreversible active Li loss upon cycling (Figure 4d). This dual‐function design enhanced the stability of the SEI and suppressed irreversible side reactions, resulting in a higher initial Coulombic efficiency of 93.04% for the preLi‐CC, compared to the 88.86% for the bare CC (Figure 4e). Over the subsequent 10 cycles, the average Coulombic efficiency for the LFP//preLi‐CC quickly stabilized at 99.90%, whereas the LFP//CC achieved only 99.86%. This improvement contributed to significantly better cycling stability for the LFP//preLi‐CC, which exhibited a capacity retention of 84% after 1000 cycles, in contrast to the 60% capacity retention observed for the LFP//CC. A large LiCoO_2_//preLi‐CC pouch cell with a size of 5 cm × 8 cm, possessing a total capacity of 100 mAh, effectively powered a wearable LED T‐shirt when integrated into the fabric (Figure 4f). Such advancements highlight the potential of preLi‐CC‐based anodes to meet the requirements of next‐generation wearable electronics.

The development of high‐performance low‐temperature Li batteries is crucial to meet power demands in cold climates. As the temperature decreases, the reaction kinetics of Li metal anodes slowdown, which can potentially lead to significantly large resistance and high cathodic/anodic over‐potential during charging and discharging processes. Conventional Li metal half‐cells fail to account for the substantial resistance of Li metal anodes during operation, which may result in unreliable performance evaluations of electrode materials and severely hinder scientific research on LIBs, particularly those designed for low‐temperature applications. Through systematic investigations into the performance of Li metal anodes in low‐temperature half‐cells, Guo et al. discovered that Li metal exhibited high SEI resistance at low temperatures, thereby inducing elevated overpotential and making it unsuitable as a counter electrode for low‐temperature batteries.^[^ ^59^ ^]^ To address this issue, they developed a promising LiC_6_@Li (0 V vs Li^+^/Li) composite electrode, which demonstrated significantly lower cathodic/anodic overpotential than Li metal under low‐temperature conditions (Figure 4g). Due to the reduced interfacial resistance of LiC_6_ (0.05 V for LiC_6_ vs 0.5 V for Li metal), lithium ions preferentially intercalated/deintercalated within the LiC_6_ compound of the composite electrode. Moreover, the Li metal rapidly replenished lithium ions in LiC_6_, enabling the LiC_6_@Li electrode to maintain an almost constant potential throughout the entire operation process. The low interfacial resistance of LiC_6_@Li can be attributed to the presence of a thin SEI layer (Figure 4h). As a result, this strategy enabled more accurate determination of Li^+^ storage potential and specific capacities for various electrode materials, including hard carbon, soft carbon, silicon carbon, Li_4_Ti_5_O_12_, and LiCoO_2_ at −20 °C (Figure 4i), indicating the viability of the LiC_6_@Li electrode as a stable and reliable counter electrode for low‐temperature half‐cell configurations.

In summary, graphitic carbon emerges as a promising host material for Li metal anodes due to its well‐aligned graphene stacking, superior electronic conductivity, and exceptional structural stability, which enable controlled Li deposition and effective dendrite suppression. However, the intrinsically low porosity of conventional graphite architectures imposes severe limitations on internal Li accommodation capacity, inevitably causing pore blockage and subsequent surface Li accumulation under high deposition loading. This may trigger parasitic reactions and rapid cycling degradation. Future research should focus on the rational design of graphite scaffolds featuring well‐defined and interconnected porous networks using sacrificial templates. Additional promising approaches include constructing vertically aligned architectures via ice‐templating or field‐assisted alignment techniques, as well as creating graphene‐based porous scaffolds through the assembly of graphene oxide or reduced graphene oxide. These tailored structures are expected to enable homogeneous Li^+^ flux distribution while providing sufficient internal space for Li accommodation. Additionally, graphitic carbon aerogels capable of actively regulating stress distributions offer a potential solution to the persistent challenge of stress‐induced Li dendrite growth.^[^ ^60^ ^]^

Nevertheless, graphite typically exhibits poorer intrinsic lithiophilicity compared to metal‐oxide‐modified carbons, which may result in elevated nucleation overpotential for Li. Strategies such as heteroatom doping or the introduction of functional groups can enhance the lithiophilicity of graphite hosts. A well‐designed graphite scaffold with low tortuosity can also facilitate rapid ion diffusion. For instance, defect‐engineered carbon nanotubes, such as defective CNT‐grown graphite, utilize defect‐rich structures to guide uniform Li plating, demonstrating exceptional cycling stability at low N/P ratios. Nevertheless, the scalability of their synthesis through chemical vapor deposition remains a challenge. Prelithiated carbon fibers combine the dual merits of Li intercalation and metal plating mechanisms while offering mechanical flexibility and self‐compensating Li reservoirs, yet their widespread application requires breakthroughs in reproducible prelithiation protocols. Promising strategies to advance hybrid anodes include: 1) developing novel electrolytes (e.g., localized high‐concentration electrolytes) to enhance compatibility with high‐capacity cathodes; 2) engineering hierarchically porous graphite frameworks with functional interlayers to guide spatially controlled lithium deposition; and 3) combining prelithiation with defect engineering to balance performance and manufacturing feasibility, ultimately enabling practical high‐energy‐density and long‐cycling hybrid anodes.

### 3.2 Disordered Carbon‐Based Hosts

Disordered carbon materials have emerged as promising host candidates for hybrid anodes in lithium‐ion batteries and lithium metal batteries owing to their heterogeneous structural features and superior electrochemical properties.^[^ ^61^, ^62^, ^63^ ^]^ These materials typically consist of a heterogeneous structure comprising graphitic domains, amorphous carbon, and mixed sp^2^–sp^3^ hybridization and exhibit remarkable advantages in electrochemical performance. Unlike conventional graphite anodes, which are limited to a specific capacity of 372 mAh g^−1^ due to Li‐ion intercalation chemistry, disordered carbons exhibit enhanced lithium storage capabilities through multiple mechanisms. These include formation of Li_2_ covalent molecules between the graphene layers with expanded interlayer spacing (>0.4 nm), lithium intercalated within carbon layers, accumulation of Li clusters stored in cavities microcavity/pores, and dual‐sided Li adsorption on isolated graphene sheets.^[^ ^64^ ^]^ Disordered carbons can be categorized into “high specific charge” and “low specific charge” carbons according to their reversible Li storage capacity. For instance, turbostratic carbons, cokes with disordered structures, and carbon blacks are classified as “low specific charge” non‐graphitic carbons, with a maximum stoichiometric factor x in Li_x_C_6_ of 0.5–0.8 arising from limited lithium sites in their wrinkled and buckled structural configurations.

When designing disordered carbon as a hybrid Li‐ion/Li‐metal anode host, several structural factors must be considered. First, a large specific surface area is essential to reduce the areal current density and homogenize the electric field distribution. Second, a well‐developed porous structure with a rational distribution of micropores, mesopores, and macropores is required to balance Li storage and electrolyte accessibility. Micropores facilitate rapid Li‐ion transport and intercalation, whereas mesopores and macropores provide space to accommodate high areal capacity Li storage. Furthermore, a robust 3D conductive network is critical to maintain efficient electron transport across the electrode, preventing localized current hotspots that could trigger dendritic growth. Additionally, mechanical flexibility is another key requirement to accommodate volume fluctuations during Li plating/stripping, thereby mitigating crack formation. Finally, the chemical stability and self‐healing capability of the solid electrolyte interphase (SEI) is important to minimize parasitic reactions and enhance cycling performance. By applying the structural versatility of disordered carbon, along with the advantages of surface defects and heteroatom doping, uniform Li deposition can be achieved, ultimately enhancing the overall battery performance.

**Figure 5:**
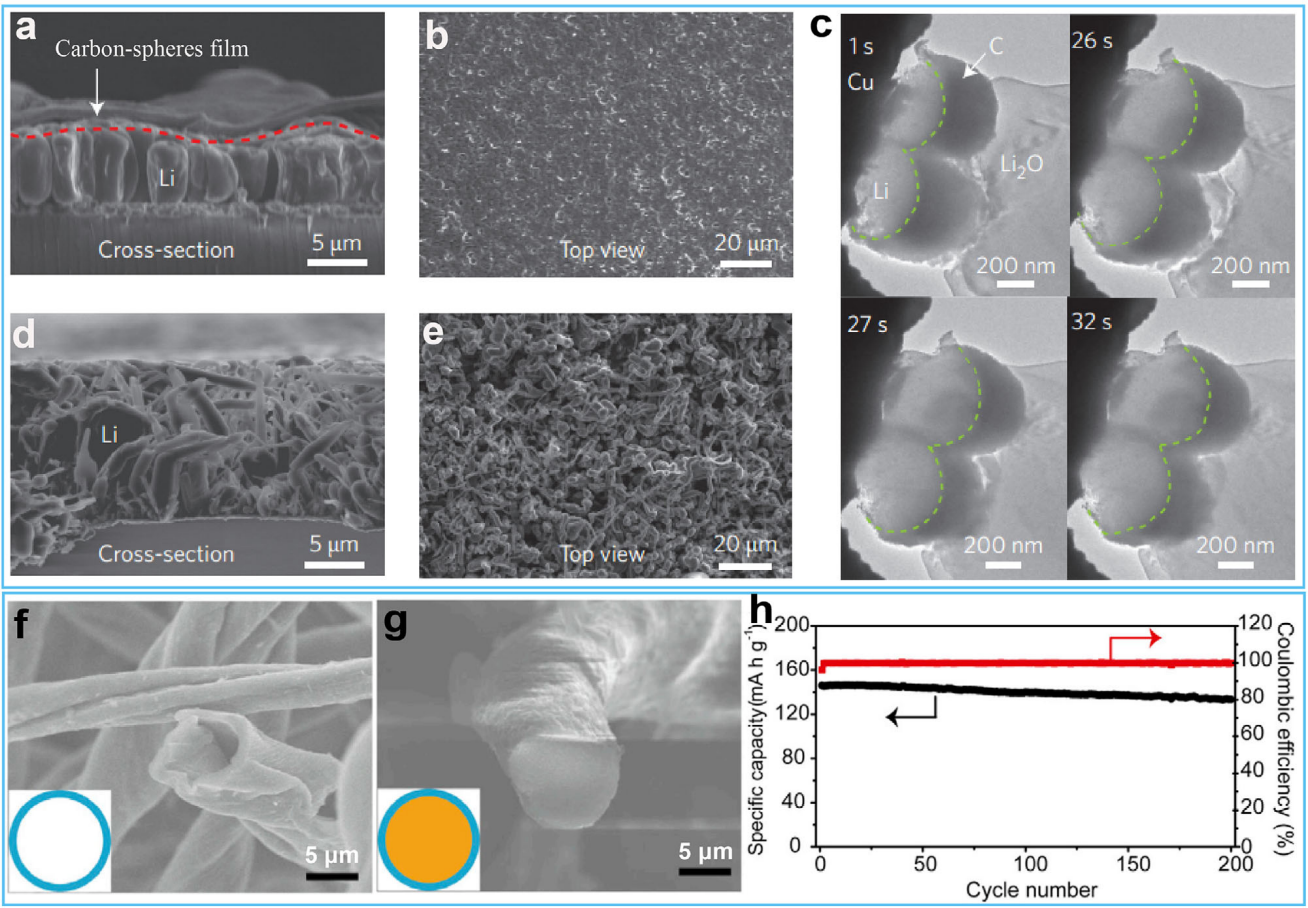
a) Cross‐sectional and b) top‐view SEM images of the hollow carbon nanosphere‐coated Cu electrode after Li deposition. c) Time‐resolved TEM images capturing Li deposition evolution on hollow carbon nanosphere‐decorated Cu wires. d) Cross‐sectional and e) top‐view SEM images of the bare Cu electrode after Li deposition. Reproduced with permission.^[^ ^65^ ^]^ Copyright 2014, Nature Publishing Group. f) Cross‐sectional SEM images of 3D‐HCFs before Li plating. g) Cross‐sectional SEM images of 3D‐HCFs after 6 mA h cm^−2^ of Li plating. h) Cycling performance of the full cell with LiFePO_4_ as the cathode and 3D‐HCFs@Li as the anode at 0.5 C. Reproduced with permission.^[^ ^67^ ^]^ Copyright 2017, Elsevier.

Thus, the artificial SEI layers and 3D carbon scaffolds based on disordered carbons are developed to enhance the electrochemical plating of Li metal. Of particular interest, a flexible, interconnected hollow amorphous carbon nanosphere coating modified Cu foil demonstrated superior Li deposition morphology and cycling stability compared to conventional Cu foil.^[^ ^65^ ^]^ Li metal began to nucleate and develop a granular morphology within the hollow carbon nanospheres on the Cu substrate, with no long filaments or dendrites emerging (**Figure** **5**a–c). In contrast, the deposition of mossy lithium directly onto the Cu electrode exhibited highly uneven growth (Figure 5d,e). This improvement was attributed to the spatially confined Li metal deposition within the nanosphere cavities and the in situ formation of a stable, self‐healing SEI layer. Although the intrinsic lithiation capacity of 0.05 mAh cm^−2^ for this carbon host was limited, its hybrid design utilized additional Li storage via intercalation mechanisms, enabling full cells assembled without pre‐deposited Li to achieve enhanced cycling performance over bare Li metal anodes. In addition to hollow carbon nanospheres, anisotropic CNTs with spatially heterogeneous structures have been engineered as artificial SEI layers for Li metal anodes.^[^ ^66^ ^]^ In such designs, the dense side of the CNT network covered with Li_3_N acted as a protective barrier to suppress parasitic electrolyte reactions, while the porous side guided dendrite‐free 3D Li deposition along the nanotube framework. However, since these CNTs lack significant Li intercalation capacity, the resulting anode operates primarily via Li metal plating/stripping, effectively constituting a LMB rather than a true hybrid system.

Carbon nanofibers (CNFs) have emerged as an ideal host material for Li metal anodes due to their distinctive 1D porous structure that spontaneously forms 3D interconnected conductive networks. These robust networks establish continuous pathways for rapid electron transport while simultaneously facilitating efficient ion diffusion through their porous structure, thereby enabling uniform Li deposition across the entire conductive matrix. The high surface‐to‐volume ratio intrinsic to CNF networks plays a pivotal role in dendrite suppression by significantly reducing local current densities. Additionally, the inherent flexibility of CNFs allows them to accommodate substantial volume changes during repeated lithium plating/stripping cycles, while their exceptional mechanical strength maintains structural integrity against the stresses induced by Li deposition.

Recent advances in Li metal anode design have demonstrated the remarkable potential of CNF‐based scaffolds for developing high‐performance 3D composite anodes through electrochemical plating strategies. A particularly noteworthy example involved the development of a lightweight, flexible, and free‐standing 3D hollow carbon fiber (3D‐HCF) architecture that enabled dual lithium storage within the inter‐fiber spaces and the hollow tubular fibers (Figure 5f,g).^[^ ^67^ ^]^ This structural design achieved exceptional electrochemical performance, with hybrid anodes exhibiting an optimal intercalation‐to‐plating capacity ratio of 1:11. Such anodes demonstrated improved cycling stability, achieving a high average Coulombic efficiency of approx≈99% over 75 cycles at a deposition areal capacity of 6 mAh cm^−2^. When paired with a practical LiFePO_4_ cathode (2 mAh cm^−2^, corresponding to an N/P ratio of 3:1), the hybrid anode configuration showed outstanding cycling stability, maintaining 91.3% of its initial capacity after 200 cycles at 0.5 C (Figure 5h). The improved stability is attributed to the capacity of lithium ions stored within the graphitic carbon matrix to gradually release and compensate for active lithium loss during cycling, thus mitigating capacity degradation. Building upon this foundation, researchers have further improved the intercalation capacity of CNF‐based scaffolds by developing graphitized carbon fiber (GCF) electrodes as multifunctional 3D current collectors.^[^ ^68^ ^]^ The GCF architecture provided an elevated intercalation capacity of 2 mAh cm^−2^, corresponding to an optimized intercalation‐to‐plating capacity ratio of 1:4. When paired with high‐areal‐capacity LiFePO_4_ cathodes (2 mA h cm^−2^), full cells incorporating GCF@Li anodes (8 mA h cm^−2^) under an N/P ratio of 4:1 demonstrated prolonged cycling stability, maintaining 80% capacity retention after 300 cycles. This performance enhancement is due to the Li_x_C_6_ phase serving as an effective Li reservoir to compensate for irreversible Li losses during cycling caused by dead Li accumulation and SEI formation. Subsequently, various efficient carbon fiber hosts have been developed for the LIB/LMB hybrid anode.^[^ ^69^ ^]^ However, these progresses have highlighted a critical challenge related to the inefficient utilization of Li metal in practical applications, which necessitates urgent attention to the development of next‐generation high‐energy‐density batteries.

**Figure 6:**
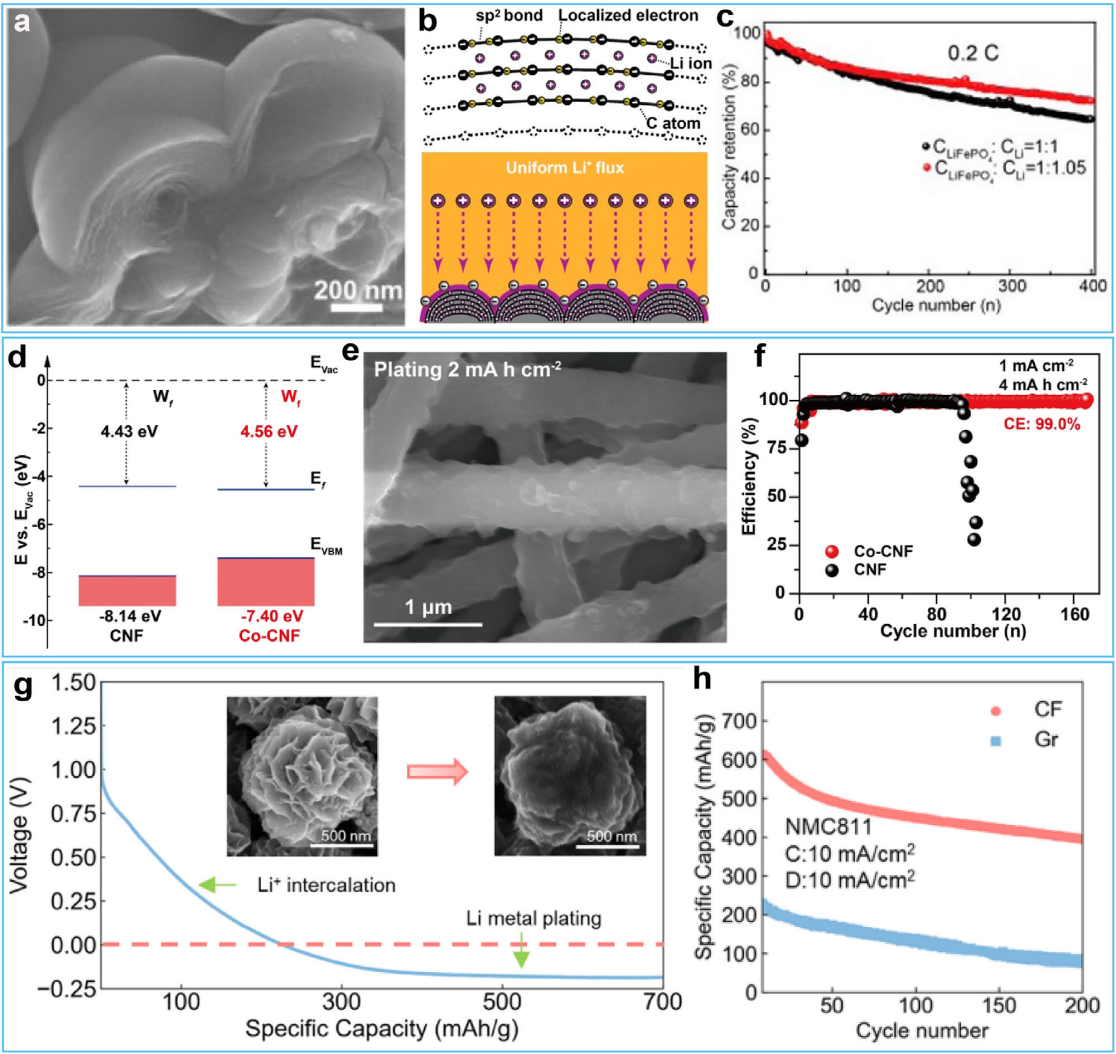
a) SEM image of the CMN. b) Schematic illustration of electron/charge distribution and Li deposition on the CMN. c) Cycling performances of the full cells based on Li‐CMN anode and LiFePO_4_ with different anode/cathode capacity ratios at 0.2 C. Reproduced with permission.^[^ ^70^ ^]^ Copyright 2017, American Chemical Society. d) Energy‐band comparison of Co‐CNF and CNF. e) SEM image of Co‐CNF morphology after Li plating. f) Coulombic efficiency comparison of Li plating/stripping cycles on Co‐CNF and CNF. Reproduced with permission.^[^ ^71^ ^]^ Copyright 2023, Wiley‐VCH. g) Electrochemical and morphological evolution of the CF electrode during charging. e) Specific capacity measured at 10 mA cm^−2^ charge/discharge current density. Reproduced with permission.^[^ ^73^ ^]^ Copyright 2022, American Chemical Society.

The utilization of metallic Li anode is one key point to decide whether the obtained Li‐metal batteries exhibit much higher energy density than LIBs. Nevertheless, this vital factor has not been comprehensively treated in current studies. In fact, much excessive amount of Li anode is commonly used to match with cathode in a Li‐metal full cell. This question is still unsolved. It is important to explore further strategies to achieve high utilization of metallic Li anode and thus guarantee a Li‐metal battery with high energy density. Carbon materials have been widely used in commercial Li‐ion batteries owing to their remarkable structural flexibility, high interface stability, and high electrochemical reversibility. Besides, carbon materials are considered promising hosts for metallic Li due to their excellent mechanical strength. Therefore, in this context, we have proposed to improve the anode performance of metal Li by using hybrid Li storage on carbon to regulate the mass/charge transfer through the Li/electrolyte interface.^[^ ^70^ ^]^ The key‐enabling technique was the use of Li‐predeposited C host with elaborately designed nanostructure consisting of eletronegative curved graphite sheets arranged to form an onion‐sphere‐like secondary architecture (**Figure** **6**a). The hybrid storage combining Li intercalation/nanoplating enhanced the surface electronegativity of C and ensured a strong binding between C and Li^+^ (Figure 6b). Particularly, the active Li^+^ preintercalated into the graphite has been intentionally reserved as an optional Li source to offset any Li loss during cycling. The result was a uniform Li^+^ flux and a stabilized Li^+^ mass/charge transfer through the Li/electrolyte interface, which suppressed the dendrite growth and brought improved anode performance. The hybrid anode showed a high Li utilization of 95% and stable plating/stripping ability, enabling a long‐life rechargeable Li metal battery (Figure 6c). While these results are promising, further investigation into transport kinetics revealed additional fundamental challenges that needed to be addressed.

To address issues of local Li^+^ hotspots and subsequent Li dendrite growth caused by the incompatibility of electron/ion transport kinetics, we developed an innovative strategy involving work function regulation of the Co‐CNF host using cobalt‐containing catalysts.^[^ ^71^ ^]^ The ultraviolet photoelectron spectroscopy (UPS), combing DFT simulations, revealed that increasing the work function drove electron transfer from carbon to cobalt, endowing Co‐CNF with a high affinity for Li^+^ and facilitating rapid catalytic reduction of Li^+^ to metallic Li (Figure 6d). In addition, the Co‐CNF host reduced the migration barrier of surface Li^+^ diffusion, allowing lithium ions to rapidly diffuse on the carbon fiber scaffolds without local aggregation or dendrite growth (Figure 6e). The optimized Li‐metal anode showed dendrite‐free Li deposition and achieved a high Coulombic efficiency of 99% (Figure 6f). The optimized Li anode also demonstrated excellent electrochemical performances in terms of long cycling life of over 2000 h with a high plating areal capacity of 4 mA h cm^−2^ in a Li|Li symmetric cell and a cycle life of 130 cycles at high cathode areal capacity of ≥ 3.0 mAh cm^−2^ and low N/P ratio of 2.5 in full pouch cells.

Following this discovery, we further proposed a rational design strategy to optimize the performance of Li metal anodes by precisely regulating the interfacial electron density and the transport of Li‐ion through the SEI film.^[^ ^72^ ^]^ Specifically, we have engineered a biomass‐derived carbon nanofiber (CNF) host by modulating the orientation of cellulose precursor fibrils, thereby reducing interfacial oxygen density. This structural modification increased the specific surface area while lowering local current density, thereby promoting the formation of a thin and uniform SEI that facilitated stable Li^+^ transport across the Li/electrolyte interface. Furthermore, the enhanced graphitization and the establishment of an interconnected conductive network elevated the surface electronegativity of the carbon scaffold, thereby ensuring continuous electron conduction. These synergistic effects enabled rapid and coupled electron/ion transport at the anode/electrolyte interface, which was critical for achieving uniform Li deposition and suppressing dendrite formation. As a result, the optimized Li/C hybrid anode demonstrated exceptional electrochemical performance, including a high initial Coulombic efficiency of 98% and extended cycling stability exceeding 150 cycles at a practical low N/P ratio of 1.44 in full‐cell configurations.

Nevertheless, the evaluation of fast‐charging anode hosts often employs testing conditions that deviate from practical battery operation, including the use of artificially low cathode loadings (<2 mAh cm^−2^), and non‐realistic anode configurations featuring either insufficient host capacity or excessively high specific capacities (>700 mAh g^−1^). Addressing these challenges, Bao and co‐workers recently designed a flower‐like, open‐pore hard carbon (CF) host for a Li‐ion/Li‐metal hybrid anode.^[^ ^73^ ^]^ Figure 6g illustrates the typical voltage profile for charging (lithiation). Notably, no significant Li nucleation overpotential was observed during the lithium plating on CF. When the voltage dropped below 0 V (vs Li^+^/Li), the thickness of the petal increased considerably, and the valleys between the intersecting “petals” began to fill in. Their design achieved an anode capacity of 700 mAh g^−1^, with 30−40% contribution from Li‐ion intercalation and 60−70% contribution from Li‐metal plating. Full cells with an N/P ratio much lower than 1 demonstrated 70% capacity retention for 200 cycles at an ultrahigh 10 mA cm^−2^ charging current (Figure 6h), highlighting the potential of hybrid anodes for high‐rate, long‐cycling applications. However, the complex fabrication process associated with this material poses significant challenges for its practical implementation.

To address dendrite growth and poor Li utilization under high current densities, Yu et al. reported a mixed ionic/electronic conducting host consisting of Zn‐embedded porous carbon nanofibers coated with NH_2_‐functionalized UiO‐66 nanoparticles (Zn/CF@NH_2_‐UiO‐66).^[^ ^74^ ^]^ The uniformly dispersed Zn acted as lithiophilic nucleation sites, while the NH_2_‐UiO‐66 coating facilitated desolvation of solvated Li^+^ ions and enhanced ion diffusion kinetics, enabling full cells with the Li–Zn/CF@NH_2_‐UiO‐66 composite anode to maintain high‐capacity retention at 2 C. In a subsequent study, they designed Sn single‐atom‐anchored subnanoporous carbon spheres for highly reversible quasi‐metallic lithium storage.^[^ ^75^ ^]^ The Sn atoms enhanced lithiophilicity and promoted confined Li deposition within internal voids, while the subnanopores restricted electrolyte penetration and mitigated side reactions, allowing full cells to maintain 80% capacity over 500 cycles at 5 C.

As an emerging class of 2D materials, transition metal carbides and carbonitrides (MXenes) have attracted growing interest for Li metal anode applications due to their high electronic conductivity, tunable interlayer structure, and abundant surface functional groups. These characteristics contribute to a strong affinity for Li, enabling MXenes to function effectively as artificial interfacial layers and host scaffolds. For interfacial stabilization, materials such as layered Ti_3_C_2_T_x_ MXene,^[^ ^76^ ^]^ BF_3_‐doped Ti_3_C,^[^ ^77^ ^]^ and Ti_3_C_2_T_x_/g–C_3_N_4_^[^ ^78^ ^]^ heterostructures have been developed to homogenize Li^+^‐ion flux and guide uniform Li deposition. Meanwhile, 3D structural designs, including MXene/metal sulfide,^[^ ^79^ ^]^ MXene/metal halides,^[^ ^80^ ^]^ flexible Ti_3_C_2_T_x_ MXene@CNF films,^[^ ^81^ ^]^ vertically aligned MXene arrays,^[^ ^82^ ^]^ low curvature MXene membranes,^[^ ^83^ ^]^ and 3D hollow MXene spheres,^[^ ^84^ ^]^ have been designed to facilitate rapid Li^+^/electron transport while accommodating volume change during cycling.

The tortuosity (*τ*) of porous carbon hosts plays a critical role in determining electrolyte accessibility and ion transport efficiency within Li anodes.^[^ ^56^, ^85^ ^]^ High‐tortuosity structures, typically featuring randomly arranged pores, result in extended diffusion pathways and increased ion migration resistance. In contrast, low‐tortuosity architectures, presented by wood‐derived carbons with vertically aligned channels, provide streamlined pathways that significantly enhance Li^+^ transport kinetics. These aligned porous networks not only facilitate rapid ion diffusion and uniform current distribution but also guide homogeneous Li deposition and alleviate volume changes, effectively suppressing dendrite formation.^[^ ^86^, ^87^ ^]^ Therefore, rational design of low‐tortuosity carbon materials is essential for developing stable, high‐capacity lithium metal anodes capable of operating reliably under practical conditions.

Carbon nanospheres demonstrate distinct advantages over carbon nanofibers as hybrid Li‐ion/Li‐metal anode hosts, offering i) an isotropic 3D architecture that mitigates Li agglomeration, ii) interconnected porosity enabling multidirectional Li^+^/e^−^ transport, iii) precisely tunable void spaces for effective volume expansion accommodation, and iv) uniform curvature that stabilizes SEI formation while suppressing dendrite growth. Recent advances in pore engineering reveal that sub‐2 nm nanopores can effectively encapsulate quasi‐metallic Li, significantly reduce parasitic reactions, and enhance electrochemical activity. This was evidenced by hard carbon hosts achieving exceptional Coulombic efficiencies of 99.7% over 240 cycles in ester electrolytes.^[^ ^88^, ^89^ ^]^ However, it is essential to strike a critical balance in pore design; while smaller nanopores (< 2 nm) enhance Li metal confinement and cycling stability, they may compromise the kinetics of Li^+^ transport and practical capacity.^[^ ^90^ ^]^ In contrast, mesoporous architectures (2–50 nm) exhibit superior Li loading capabilities while maintaining a Coulombic efficiency of 99.5%.^[^ ^91^ ^]^ These results highlight the importance of hierarchical pore optimization for the development of next‐generation hybrid anodes.

In summary, disordered carbon materials have emerged as versatile hosts for Li‐ion/Li‐metal hybrid anodes, offering structural heterogeneity that enables multiple Li storage mechanisms beyond conventional intercalation, including covalent Li_2_ formation, pore confinement, and surface adsorption. Key advantages of disordered carbon materials include tunable porosity for homogeneous Li^+^ flux, mechanical flexibility to accommodate volume changes, and defect‐rich surfaces that facilitate uniform Li deposition. Disordered carbon‐based hosts can be broadly categorized into carbon spheres and carbon fibers. 0D carbon spheres provide isotropic confinement that homogenizes local current density and promotes uniform Li nucleation across their surfaces. However, their isolated morphology may restrict long‐range electron transport. In contrast, 1D carbon fibers establish continuous conductive pathways that facilitate efficient electron transfer along the fiber axis and provide directional guidance for Li deposition, which helps mitigate dendrite formation. However, challenges remain, including limited intercalation capacity of certain carbon matrices, such as turbostratic carbons, unstable SEI, and inefficient Li utilization in full cells.

Recent strategies to address these limitations include: i) artificial SEI designs (e.g., hollow carbon nanospheres and anisotropic CNTs), which spatially confine Li plating but often lack sufficient intrinsic Li storage; ii) 3D porous CNF scaffolds (e.g., hollow/graphitized fibers), enabling dual storage of intercalation and plating with improved cycling stability, though excessive N/P ratios (>3) pose a challenge; and iii) interface‐engineered hosts (e.g., Co‐CNF and biomass‐derived CNFs), which optimize electron/ion transport kinetics, achieving a high Coulombic efficiency >99% and low N/P operation ranging from 1.44 to 2.5, yet scalability and cost barriers persist. Future research should focus on defect engineering to balance Li adsorption and SEI stability, develop dynamic hybrid mechanisms for adaptive Li storage, and advance scalable synthesis of hierarchically porous architectures. Coupled with advanced operando characterization to elucidate the lithium existence forms in disordered carbon hosts and the failure mechanisms of Li‐ion/Li‐metal hybrid anodes will facilitate broadening the applications of disordered carbons in high‐energy‐density and long‐life lithium secondary batteries.

## 4 Electrolyte Design

As a critical component of batteries, the chemical composition and physicochemical properties of electrolytes fundamentally influence the stability of electrode/electrolyte interfaces, charge transfer kinetics, and the reversibility of Li plating/stripping processes.^[^ ^92^, ^93^, ^94^ ^]^ For hybrid Li‐ion/Li‐metal anodes, electrolytes must efficiently facilitate both Li‐ion intercalation and deintercalation in graphite while ensuring uniform Li metal plating/stripping. In conventional LIBs, carbonate‐based electrolytes such as LiPF_6_ dissolved in ethylene carbonate (EC) and dimethyl carbonate (DMC) have been widely employed due to their high oxidation stability (>4.0 V) and excellent compatibility with graphite. However, the high Li activity in Li/C intercalation compounds combined with their poor thermodynamic stability leads to the decomposition of the electrolyte during initial Li^+^ intercalation and deintercalation cycles, forming a passivating SEI that is ionically conductive but electronically insulating. The formation of this SEI, along with corrosion‐like reactions involving Li_x_C_6_, results in irreversible consumption of both Li inventory and the electrolyte, contributing to significant irreversible charge losses and low initial Coulombic efficiency.^[^ ^95^ ^]^ Additionally, the repeated SEI growth with cycling increases the internal resistance of the cell and deteriorates transport dynamics. The intercalation of Li^+^ is accompanied by solvent co‐intercalation in graphite, a process known as “solvated intercalation”, which induces significant volume changes (up to 150%) that can lead to exfoliation of the graphite host and considerable performance degradation, ultimately resulting in a reduced cycle life.^[^ ^96^ ^]^

These challenges are exacerbated when Li metal anode is utilized, as carbonate electrolytes promote dendritic growth and low Coulombic efficiency (<90%) due to their high reactivity and the formation of mechanically unstable SEI.^[^ ^97^, ^98^, ^99^ ^]^ In contrast, ether‐based electrolytes, such as LiFSI in a mixture of dioxolane (DOL) and dimethoxyethane (DME), promote more uniform Li deposition and the formation of inorganic‐rich SEI layers (including LiF, Li_2_CO_3_, and Li_3_N), attributed to their low interfacial energy and weak solvation characteristics, which improve Coulombic efficiency to over 99%.^[^ ^100^, ^101^, ^102^ ^]^ However, the highest occupied molecular orbital (HOMO) energy of ether‐based solvent molecules is higher than that of saturated carbonate‐based solvent molecules.^[^ ^103^ ^]^ As a result, ether‐based electrolytes exhibit inferior oxidation resistance compared to carbonate‐based electrolytes, making it difficult to achieve charging voltages exceeding 4 V.^[^ ^104^ ^]^ Consequently, ether‐based electrolytes are unsuitable for use with high‐voltage cathodes operating above 4 V. Therefore, innovative molecular design strategies aimed at optimizing solvation structures and interfacial chemistry are essential for advancing the performance of hybrid anodes in next‐generation battery systems.

**Figure 7:**
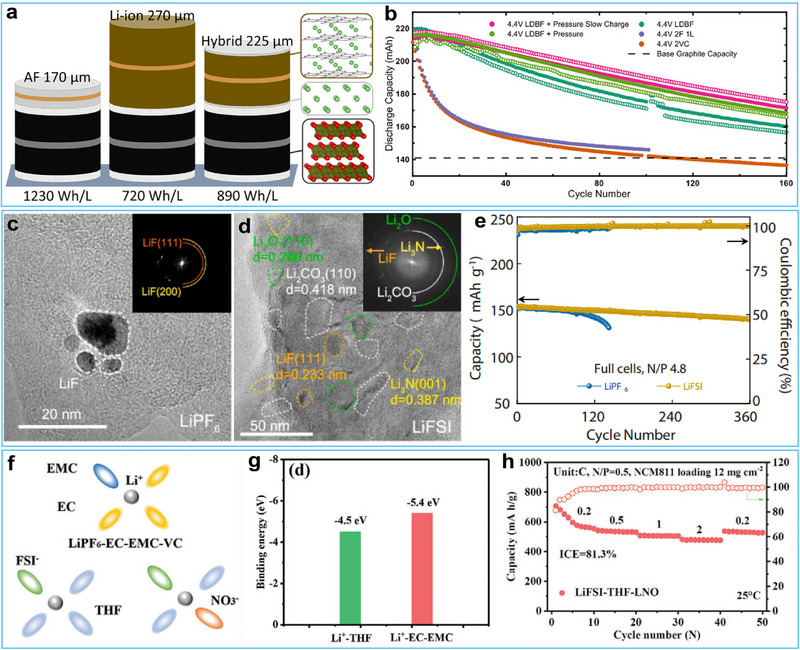
a) Schematic comparison of cell stack thickness for anode‐free Li metal (zero excess Li), conventional Li‐ion (graphite anode), and hybrid Li‐ion/Li metal configurations, scaled to electrode loadings. b) Cycling performance of the hybrid cells cycling between 3.0–4.4 V based on various electrolytes. Reproduced with permission.^[^ ^105^ ^]^ Copyright 2020, Elsevier. c, d) HRTEM characterization of SEI nanostructure after cycling in LiPF_6_ and LiFSI‐based electrolytes. e) Cycling performance of the full cells with graphite hybrid anode and LiFePO_4_ cathode in LiPF_6_ and LiFSI‐based electrolytes with N/P = 4.8 at 0.5 C. Reproduced with permission.^[^ ^106^ ^]^ Copyright 2021, Elsevier. f) Solvated structure model of the three different electrolytes. g) The calculated interaction energy between Li^+^ and different solvents. h) Rate performance of CF||NCM811 full cell in 1 m LiFSI‐THF‐0.5 wt.% LiNO_3_. Reproduced with permission.^[^ ^108^ ^]^ Copyright 2024, Wiley‐VCH.

In this regard, Dahn et al. developed a groundbreaking hybrid Li‐ion/Li‐metal anode cell design that achieved superior performance through a carefully designed dual‐salt electrolyte, composed of 1 m lithium difluoro(oxalato)borate (LiDFOB) and 0.4 m lithium tetrafluoroborate (LiBF_4_).^[^ ^105^ ^]^ The dual‐salt formulation was specifically engineered to adequately passivate Li metal while producing a flat and smooth morphology composed of large interlocked grains, addressing the inherent instability associated with Li metal plating on graphite that often led to rapid capacity fade in conventional electrolytes. The optimized electrolyte system facilitated the formation of a smooth surface with interlocked grains measuring 10–15 µm in diameter under mechanical pressure. Notably, the hybrid cell achieved a remarkable volumetric energy density of 890 Wh L^−1^ at full charge (4.4 V), surpassing the performance of conventional LIBs, which typically exhibited energy densities ≈720 Wh L^−1^ (**Figure** **7**a). Furthermore, this hybrid cell configuration maintained over 80% capacity retention over 150 cycles with a Coulombic efficiency of 99.6% (Figure 7b). The proposed system also showcased flexibility in operation, seamlessly transitioning between “Li‐ion mode” for routine energy requirements and “Li‐metal mode” for moments of heightened energy demand.

Following this discovery, Li and colleagues systematically investigated the influences of electrolytes, including lithium bis(fluorosulfonyl)imide (LiFSI) and lithium hexafluorophosphate (LiPF_6_) in the carbonate solvents, on the evolution of SEI and electrochemical performance of Li‐ion/Li metal hybrid graphite anode via in situ electrochemical atomic force microscopy.^[^ ^106^ ^]^ The SEI formed in LiPF_6_‐based electrolytes exhibited significant morphological heterogeneity, characterized by surface bulges, wrinkles, and bright nanoparticles (Figure 7c), resulting from sequential solvent decomposition followed by inorganic layer deposition that created a mesoporous organic matrix with randomly distributed inorganic components. In contrast, LiFSI‐based electrolytes promoted the preferential decomposition of salt anions to form a uniform, mechanically robust SEI with homogeneously dispersed inorganic phases within a continuous organic matrix (Figure 7d), enabling superior interfacial stability and homogeneous Li deposition. The flexibility and stability of LiFSI‐derived SEI enabled highly reversible Li plating/stripping with minimal Li consumption. This allowed the graphite anode to maintain stable cycling for 120 cycles at 3.0 mAh cm^−2^ with 92% Coulombic efficiency, showing a dramatic improvement compared to the rapid failure observed in LiPF_6_ electrolyte, which lasted only 15 cycles. When assembled into full cells consisting of a 1.75 mAh cm^−2^ LiFePO_4_ cathode paired with 3.0 mAh cm^−2^ pre‐plated Li anode, this robust interfacial chemistry sustained a reversible capacity of 140 mAh g^−1^ over 360 cycles (Figure 7e), demonstrating exceptional long‐term cycling stability. However, these reactive lithium salts tend to form a thick SEI layer on the Li surface. This layer gradually thickens during storage or under high‐capacity conditions, increasing the interfacial impedance and impairing the reversible lithium‐ion (de)intercalation and Li plating/stripping process. Therefore, further exploration of novel electrolyte designs is still needed to achieve both stability and ion transport of the SEI during high‐capacity lithium metal deposition.

Electrolyte additives play a crucial role in enhancing interfacial stability between electrodes and electrolytes in advanced battery systems. Among these additives, lithium nitrate (LiNO_3_) has been identified as particularly effective in lithium–sulfur (Li–S) batteries via effectively suppressing the shuttle of polysulfide and enhancing Li metal anode stability.^[^ ^107^ ^]^ The underlying mechanism involves the decomposition of LiNO_3_, which results in the formation of lithium nitride (Li_3_N)‐rich components within the SEI. The Li_3_N‐rich SEI not only improves Li‐ion transport kinetics due to its high ionic conductivity but also provides a high Young's modulus to inhibit Li dendrite growth. For instance, Zheng et al. demonstrated 1 m LiFSI‐THF‐0.5 wt%LiNO_3_ electrolyte ensured great enhancements toward energy and lifespan in a hybrid Li‐ion/metal anode.^[^ ^108^ ^]^ The results indicated that the introduction of LiNO_3_ increased the coordination number of anions while decreasing the coordination number of the THF solvent (Figure 7f). This alteration led to a weaker ion–solvent interaction within the THF‐driven solvation structure, resulting in a lower energy barrier for the desolvation of Li^+^ at the electrode surface (Figure 7g). Furthermore, the LiFSI‐THF‐LNO_3_ electrolyte promoted the formation of a thin and uniform solid electrolyte interphase (SEI) layer on the carbon film anode, characterized by a high content of nitrogen‐ and fluorine‐containing compounds, with an approximate thickness of 12.3 nm. The full cell, composed of an NCM811 cathode paired with a carbon film anode exhibiting a low N/P ratio of 0.5 in the LiFSI‐THF‐LNO_3_ electrolyte, delivered a capacity of 527.3 mAh g^−1^ at 25 °C and 381.5 mAh g^−1^ at −20 °C. The full cells also demonstrated favorable rate capability, achieving average capacities of 609.1, 535.4, 506.4, and 477.2 mAh g^−1^ at 0.2C, 0.5C, 1C, and 2C (1C = 500 mA g^−1^), respectively (Figure 7h). Specifically, Sun et al. conducted in situ X‐ray diffraction and Raman analysis of graphite during Li plating/stripping, demonstrating that the reversible intercalation process of Li‐ions in graphite was impeded by the formation of “dead Li” and SEI layers.^[^ ^109^ ^]^ They identified an SEI derived from LiNO_3_ that was compatible with both Li metal and graphite, which aided in maintaining the reversibility of graphite under conditions of uniform Li metal deposition.

Although LiNO_3_‐derived SEI layers improve interfacial stability through increased Li_3_N formation, their incomplete decomposition under specific conditions can result in non‐uniform SEI distribution, particularly in ether‐based electrolyte systems. This inhomogeneity is further exacerbated by competitive reduction between LiNO_3_ and other electrolyte components during SEI formation, leading to compositional inconsistency in the resulting interfacial layer. Such compromised SEI quality ultimately destabilizes graphite intercalation kinetics and diminishes reversible capacity, with these effects being particularly pronounced in hybrid anode configurations. To address these issues, Liu et al. recently introduced a soluble Mg(TFSI)_2_ electrolyte additive in a Li‐ion/Li metal hybrid anode system.^[^ ^110^ ^]^ Due to the higher standard reduction potential of Mg^2+^ (−2.37 V vs SHE) compared to Li^+^/Li (−3.04 V vs SHE), Mg^2+^ was preferentially reduced over Li, forming lithiophilic Mg nucleation seeds on graphite defect sites. These seeds guided uniform Li deposition, mitigating dendrite formation and stabilizing the anode interface, thereby significantly extending the cycle life of the hybrid batteries.

In addition, efficient electrolyte additives, such as vinyl carbonate (VC) or fluoroethylene carbonate (FEC), have been introduced into the electrolyte to reinforce the SEI film, and thus to prolong the lifetime of hybrid anodes. These additives facilitate the formation of a uniform and dense SEI rich in LiF and an elastic polymer matrix, which accommodates the volume changes during over‐lithiation and delithiation cycles.^[^ ^111^ ^]^ The mechanical integrity of the SEI is significantly improved due to the high Young's modulus of LiF, while the polymer layers serve as a selective protective barrier, facilitating Li^+^ transport while blocking larger electrolyte molecules.^[^ ^112^ ^]^ For example, Lu et al. demonstrated that FEC‐containing electrolytes enhanced hybrid battery performance at elevated temperatures by mitigating electrolyte decomposition through the formation of an in situ LiF‐rich SEI layer.^[^ ^113^ ^]^ Furthermore, Zhang et al. reported dual‐additive systems, such as 10 wt% FEC combined with 1 wt% VC, which facilitated the formation of a hybrid SEI characterized by LiF‐rich inorganic domains embedded within a polymer‐rich organic matrix. This unique structure effectively balanced ionic conductivity and mechanical resilience, thereby promoting uniform Li plating and enhancing the long‐term integrity of the SEI. The optimized SEI and 3D presetting‐void structure of the resulting graphite/Li metal hybrid anode (G@ED‐FV@Cu) achieved an impressive average Coulombic efficiency of ≈98.5% over 85 cycles. Although FEC and VC, as electrolyte additives, can significantly enhance SEI performance, there are still several critical issues: i) High concentrations of FEC (>10 wt%) can lead to increased electrolyte viscosity, subsequently reducing ionic conductivity and affecting rate performance; ii) The thicker polymer layer formed through VC polymerization may excessively hinder Li^+^ transport, particularly under low‐temperature conditions; iii) The continuous consumption of these two additives during cycling can alter the balance of electrolyte components, resulting in decreased SEI repair capability in later stages. These issues are particularly pronounced in long‐cycle operations and high‐loading electrodes.

Overall, electrolyte additives, such as LiNO_3_, VC, and FEC, play a crucial role in stabilizing hybrid Li‐ion/Li‐metal anodes by facilitating the formation of robust, multifunctional SEI layers. LiNO_3_ promotes the formation of Li_3_N‐rich SEI for improved ionic conductivity, while FEC and VC synergistically form a composite SEI comprising mechanically robust LiF domains and elastic polymer matrices. This hierarchical structure enables uniform Li plating, suppresses dendrite formation, and accommodates volume changes, ultimately resulting in high Coulombic efficiency and prolonged cycle life. However, several technical challenges remain, including the increase in electrolyte viscosity induced by FEC, the transport barriers for Li^+^ created by VC at low temperatures, and the continuous depletion of additives during extended cycling. Another fundamental issue is the incompatibility of ether‐based electrolytes with graphite anodes. Although ethers exhibit excellent stability with Li metal, they tend to co‐intercalate into graphite layers, causing structure degradation. To address these issues, future research should focus on 1) optimizing additive formulations to achieve a balance between SEI functionality and electrolyte dynamics, 2) developing novel additive systems with self‐replenishing capabilities to extend interfacial stability, and 3) developing localized high‐concentration electrolytes to preserve a stable solvation structure for reversible Li plating/stripping while minimizing graphite co‐intercalation by reducing free solvent activity. Additional promising directions include advancing in situ characterization techniques to elucidate SEI evolution mechanisms, as well as designing weakly solvating ether‐based and multi‐solvent systems. Such electrolytes can lower solvation energy, weaken the thermodynamic driving force for graphite co‐intercalation, and maintain high reductive stability against lithium metal.

## 5 Summary and Outlook

Conventional lithium‐ion batteries are inherently limited by the low theoretical capacity of graphite anodes (372 mAh g^−1^), while lithium metal batteries face significant challenges related to poor cycling stability and safety hazards due to Li dendrite growth and unstable SEI formation. Hybrid Li‐ion/Li‐metal anodes present a compelling strategy to bridge these limitations by combining the intercalation stability of host materials (e.g., graphite or disordered carbon) with the high capacity of Li metal plating. This approach not only enhances energy density but also mitigates uncontrolled Li deposition, offering a viable solution for next‐generation high‐energy storage systems. This review systematically discusses recent advances in hybrid anodes, focusing on graphite/Li, disordered carbon/Li, and electrolyte optimization, while highlighting their respective advantages, challenges, and future research directions.

Graphite, with its layered graphene structure, high electronic conductivity, exceptional structural stability, and low equilibrium potential, serves as an excellent host for Li metal, facilitating uniform nucleation and suppressing dendrite growth. However, its dense structure severely limits internal Li accommodation, leading to pore clogging and surface Li accumulation under high deposition loading. Additionally, the diffusion of Li‐ions in graphite exhibits a strong directional characteristic, as they can only be inserted perpendicularly to the end faces of the graphite crystal's *C*‐axis direction. The common orientation of graphite is parallel to the current collector, which results in longer migration pathways for Li‐ions, reducing the diffusion rate and consequently impairing high‐rate performance. To address these challenges, future efforts should focus on engineering graphite scaffolds with well‐defined, interconnected porous networks via sacrificial templates, constructing vertically aligned architectures through ice‐templating or field‐assisted alignment, as well as developing graphene‐based porous scaffolds via the assembly of graphene oxide or reduced graphene oxide. Such structures would help achieve homogeneous Li^+^ flux and provide sufficient internal space for Li accommodation.

Disordered carbons, characterized by large interlayer spacing and defect‐rich structures, exhibit enhanced Li storage capabilities through multiple mechanisms, including covalent Li_2_ formation, pore confinement, and surface adsorption. However, the Li insertion potential in disordered carbon is higher than that of graphite, which can lead to a reduction in the overall output voltage of the battery, thereby affecting its energy density. Moreover, disordered carbon typically exhibits lower lithium insertion capacity, limiting its effectiveness as a compensatory material during extended cycling. Another challenge is the high specific surface area commonly found in disordered carbons. While this can help modulate Li deposition by lowering the local current density, it may also lead to severe side reactions with the electrolyte. To address these limitations, future efforts should focus on designing disordered carbon materials with precise control of pore size distribution and tailored surface chemistry to optimize Li adsorption and stabilize the SEI. Advanced operando characterization techniques are encouraged to elucidate the Li existence forms within disordered carbon hosts and the failure mechanisms of Li‐ion/Li‐metal hybrid anodes. Multiscale modeling combining first‐principles calculations of Li nucleation behavior with continuum‐level simulations of ion transport could provide fundamental insights for host structure design. Finally, it is essential to consider scalability and manufacturing aspects early in the material development process. Future work should explore dry electrode processing for porous host materials, evaluate the environmental impact and cost‐effectiveness of synthetic routes, and develop quality control protocols to ensure porous structure uniformity in large‐format electrodes.

Electrolyte engineering is crucial for stabilizing hybrid Li‐ion/Li‐metal anodes, as it directly determines interfacial compatibility, SEI stability, and Li^+^ transport kinetics. Advanced electrolytes incorporating multiple functional additives have demonstrated synergistic effects in enhancing SEI properties, particularly in improving mechanical robustness, structural uniformity, and Li^+^ conductivity. However, these functional additives act as sacrificial components that are progressively consumed during cycling to continuously repair and regenerate degraded SEI layers, thereby maintaining interfacial integrity throughout extended cycling operations. Developing dynamic interface management systems is equally important. Unlike conventional anodes, hybrid systems undergo simultaneous intercalation and plating/stripping processes, necessitating smart interface designs. Future research should focus on stimuli‐responsive SEI components that can self‐heal and adaptively reorganize during cycling. Promising directions include designing polymer‐inorganic composite SEI layers with gradient architectures, as well as developing electrolyte additives that preferentially migrate to defect sites during cycling. Furthermore, establishing application‐oriented evaluation protocols is essential for translating laboratory advances to practical devices. It is recommended to adopt standardized testing under realistic conditions, including high areal capacity (>3 mAh cm^−2^), limited lithium excess (N/P ratio <3), and lean electrolyte (E/C ratio <5 g Ah^−1^). Beyond conventional trial‐and‐error approaches, machine learning (ML)‐assisted electrolyte design offers a transformative pathway to rapidly identify optimal formulations by predicting synergistic interactions among solvents, salts, and additives. By utilizing ML models trained on comprehensive high‐throughput experimental data and computational datasets, researchers can significantly accelerate the development of novel electrolyte systems specifically optimized for hybrid anode applications.

Overall, the practical realization of high‐performance hybrid anodes demands a multidisciplinary integration of advanced electrode architectures, precisely engineered interfaces, and scalable manufacturing processes. Future research should focus on dynamic SEI modulation to accommodate both intercalation and plating processes, in situ/operando characterization to unravel degradation mechanisms, and industry‐compatible fabrication techniques to bridge the gap between lab‐scale innovations and commercial viability. By overcoming these challenges, hybrid Li‐ion/Li‐metal anodes could unlock a new paradigm of energy storage systems, delivering exceptional energy density, prolonged cycle life, and enhanced safety for next‐generation battery applications.

## Conflict of Interest

The authors declare no conflict of interest.
